# Supplementary material for: Incidental eagle carcass detection can contribute to fatality estimation at operating wind energy facilities
Source: PLoS One. 2023 Nov 22;18(11):e0277150. doi: 10.1371/journal.pone.0277150 (PMC10664926; doi:10.1371/journal.pone.0277150)
Supplement: S1 Appendix — (DOCX) [file pone.0277150.s001.docx]

**S1 Appendix. Additional details on estimating the average probability of carcass persistence for our study sites.**

**Analysis methods**

We fit interval-censored survival regression models via GenEst [1-5] to each dataset (S1 Table A) to generate the parameter estimates required by EoA to account for persistence probability in *g*. We compared several candidate models for each study site, including different underlying persistence distributions and potential covariates. Candidate models were fit using exponential, log-logistic, lognormal, and Weibull survival distributions to characterize a broad range of persistence dynamics. Season was the only potential covariate considered in models, with the exception of Shiloh I, where there was not a sufficient sample size across seasons; thus, intercept-only models were considered for Shiloh I. We fit models that included potential covariates (where relevant) on the location and scale parameters used to define the distributions above; see Kalbfleisch and Prentice [2], Dalthorp et al. [3,4] and Therneau [5] for details about the location and scale parameterizations used for the candidate survival distributions. Model selection was performed using AICc [6] and the best-supported model was selected as the most parsimonious model within 2 AICc units of the model with the lowest AICc value. The parameter estimates of the selected model (α [shape] and β [scale], including the 95% confidence interval [CI] of β) were used as inputs in the EoA Single Class Module. See Hallingstad et al. [1] for additional details about the raptor persistence data.

**S1 Table A. Large raptor carcass persistence dataset.** Table includes raw data necessary to replicate carcass persistence analyses, from persistence trials not publically available or previously published. Data for Shiloh I was collected from nearby wind facilities in the Montezuma Hills Wind Resource Area. Data for Wild Horse was collected in previous years at Wild Horse.

| **Study Site** | **Species** | **Date Placed** | **Last Known Present (Days Since Placed)** | **First Known Absent (Days Since Placed)^a^** |
| --- | --- | --- | --- | --- |
| **Shiloh I** | Red-shouldered hawk | 2012-03-26T00:00:00Z | 27 | NA |
| **Shiloh I** | Red-tailed hawk | 2012-04-15T00:00:00Z | 28 | NA |
| **Shiloh I** | Great horned owl | 2012-05-06T00:00:00Z | 27 | NA |
| **Shiloh I** | Turkey vulture | 2012-05-12T00:00:00Z | 27 | NA |
| **Shiloh I** | Cooper's hawk | 2012-05-12T00:00:00Z | 4 | 5 |
| **Shiloh I** | Red-tailed hawk | 2012-06-11T00:00:00Z | 28 | NA |
| **Shiloh I** | Barn owl | 2012-09-17T00:00:00Z | 3 | 4 |
| **Shiloh I** | Red-shouldered hawk | 2012-10-15T00:00:00Z | 27 | NA |
| **Shiloh I** | American kestrel | 2012-11-26T00:00:00Z | 1 | 2 |
| **Shiloh I** | Barn owl | 2012-11-26T00:00:00Z | 1 | 1 |
| **Shiloh I** | Barn owl | 2013-01-02T00:00:00Z | 8 | 9 |
| **Shiloh I** | Turkey vulture | 2013-01-02T00:00:00Z | 29 | NA |
| **Shiloh I** | Great horned owl | 2013-04-01T00:00:00Z | 21 | 28 |
| **Shiloh I** | American kestrel | 2013-04-01T00:00:00Z | 2 | 3 |
| **Shiloh I** | Turkey vulture | 2013-04-01T00:00:00Z | 28 | NA |
| **Shiloh I** | Northern harrier | 2013-05-06T00:00:00Z | 6 | 7 |
| **Shiloh I** | Red-tailed hawk | 2013-05-06T00:00:00Z | 28 | NA |
| **Shiloh I** | Osprey | 2013-06-03T00:00:00Z | 27 | NA |
| **Shiloh I** | American kestrel | 2013-06-03T00:00:00Z | 1 | 2 |
| **Shiloh I** | Red-tailed hawk | 2013-07-08T00:00:00Z | 28 | NA |
| **Shiloh I** | Turkey vulture | 2013-07-08T00:00:00Z | 28 | NA |
| **Shiloh I** | Great horned owl | 2013-08-05T00:00:00Z | 1 | 1 |
| **Shiloh I** | Barn owl | 2013-08-05T00:00:00Z | 5 | 6 |
| **Shiloh I** | Red-tailed hawk | 2013-09-03T00:00:00Z | 34 | NA |
| **Shiloh I** | Turkey vulture | 2013-10-07T00:00:00Z | 28 | NA |
| **Shiloh I** | American kestrel | 2013-10-07T00:00:00Z | 3 | 4 |
| **Shiloh I** | Red-tailed hawk | 2022-03-12T00:00:00Z | 14 | NA |
| **Shiloh I** | Turkey vulture | 2022-09-12T00:00:00Z | 14 | NA |
| **Shiloh I** | Turkey vulture | 2022-09-12T00:00:00Z | 14 | NA |
| **Shiloh I** | Turkey vulture | 2022-09-12T00:00:00Z | 14 | NA |
| **Shiloh I** | Red-tailed hawk | 2022-12-12T00:00:00Z | 14 | NA |
| **Shiloh I** | Red-tailed hawk | 2022-12-12T00:00:00Z | 14 | NA |
| **Shiloh I** | Red-tailed hawk | 2022-12-12T00:00:00Z | 12 | 12 |
| **Shiloh I** | Red-tailed hawk | 2022-01-13T00:00:00Z | 14 | NA |
| **Shiloh I** | Turkey vulture | 2022-01-13T00:00:00Z | 14 | NA |
| **Wild Horse** | Barred owl | 2020-01-03T00:00:00Z | 90 | NA |
| **Wild Horse** | Barred owl | 2020-01-03T00:00:00Z | 90 | NA |
| **Wild Horse** | Barred owl | 2020-01-03T00:00:00Z | 90 | NA |
| **Wild Horse** | Barred owl | 2020-01-03T00:00:00Z | 3 | 3 |
| **Wild Horse** | Barred owl | 2020-01-03T00:00:00Z | 90 | NA |
| **Wild Horse** | Barred owl | 2020-01-03T00:00:00Z | 75 | 90 |
| **Wild Horse** | Barred owl | 2020-01-03T00:00:00Z | 5 | NA |
| **Wild Horse** | Barred owl | 2020-01-03T00:00:00Z | 5 | 10 |
| **Wild Horse** | Barred owl | 2020-01-03T00:00:00Z | 90 | NA |
| **Wild Horse** | Barred owl | 2020-01-03T00:00:00Z | 90 | NA |
| **Wild Horse** | Barred owl | 2020-01-03T00:00:00Z | 5 | 10 |
| **Wild Horse** | Barred owl | 2020-01-03T00:00:00Z | 32 | 48 |
| **Wild Horse** | Barred owl | 2020-01-03T00:00:00Z | 5 | 10 |
| **Wild Horse** | Barred owl | 2020-01-03T00:00:00Z | 75 | 90 |
| **Wild Horse** | Barred owl | 2020-01-03T00:00:00Z | 5 | 10 |
| **Wild Horse** | Barred owl | 2020-01-03T00:00:00Z | 21 | 32 |
| **Wild Horse** | Barred owl | 2020-01-03T00:00:00Z | 3 | 3 |
| **Wild Horse** | Barred owl | 2020-01-03T00:00:00Z | 60 | 75 |
| **Wild Horse** | Barred owl | 2020-01-03T00:00:00Z | 90 | NA |
| **Wild Horse** | Barred owl | 2020-01-03T00:00:00Z | 90 | NA |
| **Wild Horse** | Barred owl | 2020-09-03T00:00:00Z | 90 | NA |
| **Wild Horse** | Barred owl | 2020-09-03T00:00:00Z | 43 | 60 |
| **Wild Horse** | Barred owl | 2020-09-03T00:00:00Z | 90 | NA |
| **Wild Horse** | Barred owl | 2020-09-03T00:00:00Z | 90 | NA |
| **Wild Horse** | Barred owl | 2020-09-03T00:00:00Z | 90 | NA |
| **Wild Horse** | Barred owl | 2020-09-03T00:00:00Z | 12 | 20 |
| **Wild Horse** | Barred owl | 2020-09-03T00:00:00Z | 90 | NA |
| **Wild Horse** | Barred owl | 2020-09-03T00:00:00Z | 29 | 43 |
| **Wild Horse** | Barred owl | 2020-09-03T00:00:00Z | 29 | 43 |
| **Wild Horse** | Barred owl | 2020-09-03T00:00:00Z | 5 | 12 |
| **Wild Horse** | Barred owl | 2020-09-03T00:00:00Z | 5 | 12 |
| **Wild Horse** | Barred owl | 2020-09-03T00:00:00Z | 1 | 5 |
| **Wild Horse** | Barred owl | 2020-09-03T00:00:00Z | 29 | 43 |
| **Wild Horse** | Barred owl | 2020-09-03T00:00:00Z | 60 | 75 |
| **Wild Horse** | Barred owl | 2020-09-03T00:00:00Z | 90 | NA |
| **Wild Horse** | Barred owl | 2020-09-03T00:00:00Z | 12 | 20 |
| **Wild Horse** | Barred owl | 2020-09-03T00:00:00Z | 1 | 1 |
| **Wild Horse** | Barred owl | 2020-09-03T00:00:00Z | 5 | 12 |
| **Wild Horse** | Barred owl | 2020-09-03T00:00:00Z | 90 | NA |
| **Wild Horse** | Barred owl | 2020-09-03T00:00:00Z | 5 | 12 |
| **Wild Horse** | Barn owl | 2016-05-14T00:00:00Z | 86 | 98 |
| **Wild Horse** | Red-tailed hawk | 2016-05-14T00:00:00Z | 122 | NA |
| **Wild Horse** | Barn owl | 2016-05-14T00:00:00Z | 86 | 98 |
| **Wild Horse** | Red-tailed hawk | 2016-05-14T00:00:00Z | 122 | NA |
| **Wild Horse** | Barn owl | 2016-05-14T00:00:00Z | 86 | 98 |
| **Wild Horse** | Barn owl | 2016-05-14T00:00:00Z | 86 | 98 |
| **Wild Horse** | Red-tailed hawk | 2016-05-14T00:00:00Z | 98 | 118 |
| **Wild Horse** | Red-tailed hawk | 2016-05-14T00:00:00Z | 122 | NA |
| **Wild Horse** | Great horned owl | 2016-05-14T00:00:00Z | 122 | NA |
| **Wild Horse** | Barn owl | 2016-05-14T00:00:00Z | 122 | NA |
| **Wild Horse** | Barn owl | 2016-05-14T00:00:00Z | 86 | 98 |
| **Wild Horse** | Red-tailed hawk | 2016-05-14T00:00:00Z | 122 | NA |
| **Wild Horse** | Barn owl | 2016-05-14T00:00:00Z | 34 | 46 |
| **Wild Horse** | Red-tailed hawk | 2016-05-14T00:00:00Z | 34 | 46 |
| **Wild Horse** | Barn owl | 2016-05-14T00:00:00Z | 122 | NA |
| **Wild Horse** | Prairie falcon | 2016-05-14T00:00:00Z | 122 | NA |
| **Wild Horse** | Barn owl | 2016-05-14T00:00:00Z | 98 | 118 |
| **Wild Horse** | Barn owl | 2016-05-14T00:00:00Z | 86 | 98 |

^a^NA = not applicable

**Results**

The best-supported models of raptor persistence for each study site can be found in S1 Table B. The best-supported models for Marble River and Wild Horse included a season covariate, while the models for all other sites were intercept-only. Median carcass persistence and average probability of persistence estimates (based on a 30-day search interval) for the datasets most representative of each of the study sites are presented in S1 Table C. Persistence trial data from all 4 seasons were available to correspond with our incidental detection trials, with the exception of Wild Horse where summer data were not available. Instead, the fall estimate of persistence at Wild Horse was used for summer as a conservative proxy since it was the lowest of the 3 represented seasons.

Model parameter estimates and 95% confidence intervals of the β parameter used in EoA to inform the incidental *g*’s can be found in S1 Table D.

**S1 Table B. The best-supported survival regression models of large raptor carcass persistence for each study site.**

| **Trial Site** | **Incidental *g* Study Site** | **Distribution** | **Location Parameter** | **Scale Parameter^a^** | **Number of Parameters** | **Sample Size** | **AICc^b^** | **Δ AICc^c^** |
| --- | --- | --- | --- | --- | --- | --- | --- | --- |
| **Hale Wind Farm** | **Frontier I** | Exponential | No covariates | NA | 1 | 40 | 121.28 | 0^d^ |
| **Hale Wind Farm** | **Frontier I** | Weibull | No covariates | No covariates | 2 | 40 | 122.45 | 1.17 |
| **Hale Wind Farm** | **Frontier I** | Exponential | Season | NA | 4 | 40 | 122.58 | 1.3 |
| **Hale Wind Farm** | **Frontier I** | Loglogistic | No covariates | No covariates | 2 | 40 | 122.72 | 1.44 |
| **Hale Wind Farm** | **Frontier I** | Lognormal | No covariates | No covariates | 2 | 40 | 122.9 | 1.62 |
| **Hale Wind Farm** | **Frontier I** | Weibull | Season | No covariates | 5 | 40 | 124.55 | 3.27 |
| **Hale Wind Farm** | **Frontier I** | Loglogistic | Season | No covariates | 5 | 40 | 125.47 | 4.19 |
| **Hale Wind Farm** | **Frontier I** | Lognormal | Season | No covariates | 5 | 40 | 126.44 | 5.16 |
| **Hale Wind Farm** | **Frontier I** | Weibull | No covariates | Season | 5 | 40 | 128.76 | 7.48 |
| **Hale Wind Farm** | **Frontier I** | Loglogistic | No covariates | Season | 5 | 40 | 129.69 | 8.41 |
| **Hale Wind Farm** | **Frontier I** | Lognormal | No covariates | Season | 5 | 40 | 130.05 | 8.77 |
| **Hale Wind Farm** | **Frontier I** | Weibull | Season | Season | 8 | 40 | 131.96 | 10.68 |
| **Hale Wind Farm** | **Frontier I** | Loglogistic | Season | Season | 8 | 40 | 132.35 | 11.07 |
| **Hale Wind Farm** | **Frontier I** | Lognormal | Season | Season | 8 | 40 | 132.52 | 11.24 |
| **Arkwright Summit Wind Farm** | **Marble River** | Weibull | Season | Season | 8 | 40 | 153.9 | 0^d^ |
| **Arkwright Summit Wind Farm** | **Marble River** | Exponential | Season | NA | 4 | 40 | 156.24 | 2.34 |
| **Arkwright Summit Wind Farm** | **Marble River** | Weibull | No covariates | Season | 5 | 40 | 157.59 | 3.69 |
| **Arkwright Summit Wind Farm** | **Marble River** | Exponential | No covariates | NA | 1 | 40 | 157.61 | 3.71 |
| **Arkwright Summit Wind Farm** | **Marble River** | Weibull | Season | No covariates | 5 | 40 | 157.65 | 3.75 |
| **Arkwright Summit Wind Farm** | **Marble River** | Weibull | No covariates | No covariates | 2 | 40 | 159.81 | 5.91 |
| **Arkwright Summit Wind Farm** | **Marble River** | Lognormal | Season | No covariates | 5 | 40 | 160.86 | 6.96 |
| **Arkwright Summit Wind Farm** | **Marble River** | Loglogistic | Season | No covariates | 5 | 40 | 161.23 | 7.33 |
| **Arkwright Summit Wind Farm** | **Marble River** | Lognormal | Season | Season | 8 | 40 | 161.56 | 7.66 |
| **Arkwright Summit Wind Farm** | **Marble River** | Loglogistic | Season | Season | 8 | 40 | 162.2 | 8.3 |
| **Arkwright Summit Wind Farm** | **Marble River** | Lognormal | No covariates | No covariates | 2 | 40 | 165.81 | 11.91 |
| **Arkwright Summit Wind Farm** | **Marble River** | Loglogistic | No covariates | No covariates | 2 | 40 | 167.2 | 13.3 |
| **Arkwright Summit Wind Farm** | **Marble River** | Lognormal | No covariates | Season | 5 | 40 | 168.11 | 14.21 |
| **Arkwright Summit Wind Farm** | **Marble River** | Loglogistic | No covariates | Season | 5 | 40 | 169.17 | 15.27 |
| **Milford Wind Corridor** | **Mountain Wind I and II** | Exponential | No covariates | NA | 1 | 39 | 154.38 | 0^d^ |
| **Milford Wind Corridor** | **Mountain Wind I and II** | Lognormal | No covariates | No covariates | 2 | 39 | 155.08 | 0.7 |
| **Milford Wind Corridor** | **Mountain Wind I and II** | Loglogistic | No covariates | No covariates | 2 | 39 | 155.9 | 1.52 |
| **Milford Wind Corridor** | **Mountain Wind I and II** | Weibull | No covariates | No covariates | 2 | 39 | 156.41 | 2.03 |
| **Milford Wind Corridor** | **Mountain Wind I and II** | Lognormal | No covariates | Season | 5 | 39 | 156.57 | 2.19 |
| **Milford Wind Corridor** | **Mountain Wind I and II** | Exponential | Season | NA | 4 | 39 | 156.59 | 2.21 |
| **Milford Wind Corridor** | **Mountain Wind I and II** | Loglogistic | No covariates | Season | 5 | 39 | 157.37 | 2.99 |
| **Milford Wind Corridor** | **Mountain Wind I and II** | Lognormal | Season | No covariates | 5 | 39 | 157.98 | 3.6 |
| **Milford Wind Corridor** | **Mountain Wind I and II** | Weibull | No covariates | Season | 5 | 39 | 158.69 | 4.31 |
| **Milford Wind Corridor** | **Mountain Wind I and II** | Loglogistic | Season | No covariates | 5 | 39 | 158.75 | 4.37 |
| **Milford Wind Corridor** | **Mountain Wind I and II** | Weibull | Season | No covariates | 5 | 39 | 159.17 | 4.79 |
| **Milford Wind Corridor** | **Mountain Wind I and II** | Lognormal | Season | Season | 8 | 39 | 161.33 | 6.95 |
| **Milford Wind Corridor** | **Mountain Wind I and II** | Loglogistic | Season | Season | 8 | 39 | 162.13 | 7.75 |
| **Milford Wind Corridor** | **Mountain Wind I and II** | Weibull | Season | Season | 8 | 39 | 163.07 | 8.69 |
| **El Cabo Wind Farm** | **Pinyon Pines I and II** | Weibull | No covariates | No covariates | 2 | 40 | 121.52 | 0 |
| **El Cabo Wind Farm** | **Pinyon Pines I and II** | Loglogistic | No covariates | No covariates | 2 | 40 | 121.66 | 0.14 |
| **El Cabo Wind Farm** | **Pinyon Pines I and II** | Lognormal | No covariates | No covariates | 2 | 40 | 121.84 | 0.32 |
| **El Cabo Wind Farm** | **Pinyon Pines I and II** | Exponential | No covariates | NA | 1 | 40 | 122.95 | 1.43^d^ |
| **El Cabo Wind Farm** | **Pinyon Pines I and II** | Weibull | Season | No covariates | 5 | 40 | 125.38 | 3.86 |
| **El Cabo Wind Farm** | **Pinyon Pines I and II** | Lognormal | Season | No covariates | 5 | 40 | 125.45 | 3.93 |
| **El Cabo Wind Farm** | **Pinyon Pines I and II** | Loglogistic | Season | No covariates | 5 | 40 | 125.66 | 4.14 |
| **El Cabo Wind Farm** | **Pinyon Pines I and II** | Exponential | Season | NA | 4 | 40 | 126.17 | 4.65 |
| **El Cabo Wind Farm** | **Pinyon Pines I and II** | Weibull | No covariates | Season | 5 | 40 | 126.41 | 4.89 |
| **El Cabo Wind Farm** | **Pinyon Pines I and II** | Lognormal | No covariates | Season | 5 | 40 | 127.43 | 5.91 |
| **El Cabo Wind Farm** | **Pinyon Pines I and II** | Loglogistic | No covariates | Season | 5 | 40 | 127.57 | 6.05 |
| **El Cabo Wind Farm** | **Pinyon Pines I and II** | Weibull | Season | Season | 8 | 40 | 133.98 | 12.46 |
| **El Cabo Wind Farm** | **Pinyon Pines I and II** | Lognormal | Season | Season | 8 | 40 | 134.21 | 12.69 |
| **El Cabo Wind Farm** | **Pinyon Pines I and II** | Loglogistic | Season | Season | 8 | 40 | 134.37 | 12.85 |
| **Montezuma Hills Wind Resource Area** | **Shiloh I** | Lognormal | No covariates | No covariates | 2 | 35 | 117.7 | 0^d^ |
| **Montezuma Hills Wind Resource Area** | **Shiloh I** | Loglogistic | No covariates | No covariates | 2 | 35 | 118.26 | 0.56 |
| **Montezuma Hills Wind Resource Area** | **Shiloh I** | Weibull | No covariates | No covariates | 2 | 35 | 118.85 | 1.15 |
| **Montezuma Hills Wind Resource Area** | **Shiloh I** | Exponential | No covariates | NA | 1 | 35 | 123.02 | 5.32 |
| **Wild Horse** | **Wild Horse** | Weibull | No covariates | Season | 4 | 58 | 226.93 | 0^d^ |
| **Wild Horse** | **Wild Horse** | Loglogistic | Season | Season | 6 | 58 | 230.27 | 3.34 |
| **Wild Horse** | **Wild Horse** | Weibull | Season | Season | 6 | 58 | 230.75 | 3.82 |
| **Wild Horse** | **Wild Horse** | Lognormal | Season | Season | 6 | 58 | 230.85 | 3.92 |
| **Wild Horse** | **Wild Horse** | Loglogistic | Season | No covariates | 4 | 58 | 240.27 | 13.34 |
| **Wild Horse** | **Wild Horse** | Exponential | Season | NA | 3 | 58 | 240.3 | 13.37 |
| **Wild Horse** | **Wild Horse** | Weibull | No covariates | No covariates | 2 | 58 | 240.33 | 13.4 |
| **Wild Horse** | **Wild Horse** | Weibull | Season | No covariates | 4 | 58 | 240.71 | 13.78 |
| **Wild Horse** | **Wild Horse** | Lognormal | Season | No covariates | 4 | 58 | 240.88 | 13.95 |
| **Wild Horse** | **Wild Horse** | Exponential | No covariates | NA | 1 | 58 | 241.3 | 14.37 |
| **Wild Horse** | **Wild Horse** | Loglogistic | No covariates | No covariates | 2 | 58 | 242.5 | 15.57 |
| **Wild Horse** | **Wild Horse** | Lognormal | No covariates | No covariates | 2 | 58 | 243.56 | 16.63 |

^a^ NA = not applicable

^b^AICc is corrected Akaike’s Information Criterion

^c^Δ AICc is the difference between the models.

^d^We used this model in the analysis.

**S1 Table C. Sources of raptor persistence data and summary of modeled results.** Sources of raptor persistence data and summary of modeled results for data used to adjust overall probability of incidental detection (Incidental *g*) for raptor persistence.

| **Incidental Detection Study Site, State, and Predominant Land Cover(s)** | **Raptor Persistence Data Source, State, and Predominant Land Cover** | **Number of Raptor Carcasses** | **Median Persistence Time (days)** | **Average Probability of Persistence (30 days)** |
| --- | --- | --- | --- | --- |
| **Mountain Wind I and II (WY), shrub/scrub and grassland** | **Milford (UT), shrub/scrub [1]** | 39 | 78.60 | 0.88 |
| **Pinyon Pines I and II (CA), shrub/scrub** | **El Cabo (NM), grassland [1]** | 40 | 65.53 | 0.86 |
| **Shiloh I (CA), grassland and cropland** | **Montezuma Hills Wind Resource Area facilities (CA), cropland (S8 Table)** | 35 | 50.96 | 0.70 |
| **Wild Horse (WA), shrub/scrub** | **Wild Horse (WA), shrub/scrub (S8 Table)** | 58 | 67.79 (fall)–112.59 (spring) | 0.77 (fall)–0.99 (spring) |
| **Frontier I (OK), cropland** | **Hale (TX), cropland [1]** | 40 | 170.12 | 0.94 |
| **Marble River (NY), forest and cropland** | **Arkwright (NY), forest [1]** | 40 | 4.82 (spring)–40.43 (summer) | 0.33 (spring)–0.94 (summer) |

**S1 Table D. Model distribution, and required parameter estimates and 95% confidence intervals of the survival regression parameter(s) used in Evidence of Absence to inform the overall probability of incidental detection (incidental *g*) estimates.**

| **Trial Site** | **Incidental *g* Study Site** | **Season** | **Sample Size** | **Distribution** | **Pda (Alpha) Parameter** | **Pdb (Beta) Parameter** | **Pdb (Beta) Parameter Lower Limit** | **Pdb (Beta) Parameter Upper Limit** |
| --- | --- | --- | --- | --- | --- | --- | --- | --- |
| **Hale Wind Farm** | **Frontier I** | All | 40 | exponential | - | 106.911 | 65.497 | 174.513 |
| **Arkwright Summit Wind Farm** | **Marble River** | Fall | 10 | Weibull | 1.37 | 17.013 | 10.392 | 27.855 |
| **Arkwright Summit Wind Farm** | **Marble River** | Spring | 10 | Weibull | 0.553 | 9.356 | 2.389 | 36.598 |
| **Arkwright Summit Wind Farm** | **Marble River** | Summer | 10 | Weibull | 3.096 | 45.513 | 36.892 | 56.149 |
| **Arkwright Summit Wind Farm** | **Marble River** | Winter | 10 | Weibull | 0.956 | 34.64 | 16.151 | 74.292 |
| **Milford Wind Corridor** | **Mountain Wind I and II** | All | 39 | exponential | - | 113.409 | 68.375 | 188.105 |
| **El Cabo Wind Farm** | **Pinyon Pines I and II** | All | 40 | exponential | - | 94.538 | 58.792 | 152.17 |
| **Montezuma Hills Wind Resource Area** | **Shiloh I** | All | 35 | lognormal | 7.36 | 3.931 | 2.563 | 5.3 |
| **Wild Horse** | **Wild Horse** | Fall | 20 | Weibull | 0.584 | 126.976 | 104.063 | 154.934 |
| **Wild Horse** | **Wild Horse** | Spring | 18 | Weibull | 3.049 | 126.976 | 104.063 | 154.934 |
| **Wild Horse** | **Wild Horse** | Winter | 20 | Weibull | 0.588 | 126.976 | 104.063 | 154.934 |

**References for S1 Appendix**

1. Hallingstad E, Riser-Espinoza D, Brown S, Rabie P, Haddock J, Kosciuch K. Game bird carcasses are less persistent than raptor carcasses, but can predict raptor persistence dynamics. PLoS ONE 2023; 18: e0279997. doi: 10.1371/journal.pone.0279997.
2. Kalbfleisch JD, Prentice RL. The statistical analysis of failure time data. Hoboken, New Jersey: John Wiley & Sons; 2002.
3. Dalthorp DH, Madsen L, Huso MM, Rabie P, Wolpert R, Studyvin J, et al. GenEst statistical models—a generalized estimator of mortality. U.S. Geological Survey Techniques and Methods. doi: 10.3133/tm7A2. 2018 [cited 2022 Aug 22]. Available from: https://pubs.usgs.gov/tm/7a2/tm7a2.pdf
4. Dalthorp DH, Simonis J, Madsen L, Huso MM, Rabie P, Mintz JM, et al.. GenEst: generalized mortality estimator. Version 1.4.6 [software]. 2021 June 17 [cited 2022 Aug 22]. Available from: https://CRAN.R-project.org/package=GenEst
5. Therneau T. A package for survival analysis in R. Version 3.2-9. 2021. March 14 [cited 2022 Oct 19]. Available from: http://CRAN.R-project.org/package=survival
6. Burnham KP, Anderson DR. Model selection and multimodel interface: a practical information-theoretical approach. 2nd ed. New York: Springer-Verlag New York, Inc.; 2010.
