# Supplementary material for: Incidental eagle carcass detection can contribute to fatality estimation at operating wind energy facilities
Source: PLoS One. 2023 Nov 22;18(11):e0277150. doi: 10.1371/journal.pone.0277150 (PMC10664926; doi:10.1371/journal.pone.0277150)
Supplement: S1 Table — Complexity classes by season during detection trials conducted at the study sites from June 27, 2021, through July 14, 2022. (DOCX) [file pone.0277150.s002.docx]

**S2 Table. Percent coverage of viewshed complexity classes within the 100-m radius search areas centered on wind turbine towers.** Complexity classes by season during detection trials conducted at the study sites from June 27, 2021, through July 14, 2022.

| **Study Site** | **Season** | **Viewshed Complexity Class** | | | |
| --- | --- | --- | --- | --- | --- |
|  |  | **Percent Low** | **Percent Moderate** | **Percent High** | **Percent Unviewable/ Unsearchable^a^** |
| **Frontier** | Spring | 40.1 | 45.6 | 0 | 14.3 |
|  | Summer | 42.6 | 0 | 0 | 57.4 |
|  | Fall | 85.7 | 0 | 0 | 14.3 |
|  | Winter | 85.7 | 0 | 0 | 14.3 |
| **Marble River** | Spring | 6.9 | 6.0 | 8.7 | 78.5 |
|  | Summer | 6.9 | 3.4 | 8.7 | 81.0 |
|  | Fall | 9.4 | 3.4 | 8.7 | 78.5 |
|  | Winter | 0 | 0 | 0 | 100 |
| **Mountain Wind I and II** | Spring | 39.0 | 57.2 | 1.2 | 2.7 |
|  | Summer | 39.0 | 57.2 | 1.2 | 2.7 |
|  | Fall | 39.0 | 57.2 | 1.2 | 2.7 |
|  | Winter | 0 | 0 | 0 | 100 |
| **Pinyon Pines I and II** | Spring | 13.0 | 16.3 | 70.7 | 0.1 |
|  | Summer | 13.0 | 16.3 | 70.7 | 0.1 |
|  | Fall | 13.0 | 16.3 | 70.7 | 0.1 |
|  | Winter | 13.0 | 16.3 | 70.7 | 0.1 |
| **Shiloh I** | Spring | 40.0 | 33.2 | 0 | 26.8 |
|  | Summer | 40.0 | 0 | 0 | 60.0 |
|  | Fall | 73.2 | 0 | 0 | 26.8 |
|  | Winter | 40.0 | 33.2 | 0 | 26.8 |
| **Wild Horse** | Spring | 11.9 | 74.8 | 3.2 | 10.1 |
|  | Summer | 11.9 | 74.8 | 3.2 | 10.1 |
|  | Fall | 11.9 | 74.8 | 3.2 | 10.1 |
|  | Winter | 11.9 | 74.8 | 3.2 | 10.1 |

^a^Sums may not total 100 percent, due to rounding.
