# Supplementary material for: Incidental eagle carcass detection can contribute to fatality estimation at operating wind energy facilities
Source: PLoS One. 2023 Nov 22;18(11):e0277150. doi: 10.1371/journal.pone.0277150 (PMC10664926; doi:10.1371/journal.pone.0277150)
Supplement: S2 Table — Trial results among viewshed complexity classes during incidental detection trials conducted at the study sites from June 27, 2021, through July 14, 2022. (DOCX) [file pone.0277150.s003.docx]

**S3 Table. Incidental detection trial results among viewshed complexity classes.** Trial results among viewshed complexity classes during incidental detection trials conducted at the study sites from June 27, 2021, through July 14, 2022.

| **Study Site** | **Viewshed Complexity** | **Placed** | **Available** | **Found** | **Detection** |
| --- | --- | --- | --- | --- | --- |
| **Frontier I** | Low | 114 | 92 | 53 | 0.58 |
| **Marble River** | Low | 78 | 56 | 50 | 0.89 |
| **Mountain Wind I and II** | Low | 71 | 71 | 60 | 0.85 |
| **Pinyon Pines I and II** | Low | 67 | 64 | 37 | 0.58 |
| **Shiloh I** | Low | 129 | 127 | 101 | 0.80 |
| **Wild Horse** | Low | 128 | 126 | 39 | 0.31 |
| **Frontier I** | Moderate | 26 | 22 | 2 | 0.09 |
| **Marble River** | Moderate | 27 | 22 | 17 | 0.77 |
| **Mountain Wind I and II** | Moderate | 63 | 63 | 30 | 0.48 |
| **Pinyon Pines I and II** | Moderate | 73 | 72 | 18 | 0.25 |
| **Shiloh I** | Moderate | 30 | 28 | 12 | 0.43 |
| **Wild Horse** | Moderate | 42 | 42 | 10 | 0.24 |
| **Frontier I** | High | 37 | 26 | 8 | 0.31 |
| **Marble River** | High | 11 | 8 | 0 | 0 |
| **Mountain Wind I and II** | High | 10 | 10 | 1 | 0.10 |
| **Pinyon Pines I and II** | High | 52 | 52 | 4 | 0.08 |
| **Shiloh I** | High | 32 | 31 | 2 | 0.06 |
| **Wild Horse** | High | 6 | 6 | 0 | 0 |
