# Supplementary material for: Incidental eagle carcass detection can contribute to fatality estimation at operating wind energy facilities
Source: PLoS One. 2023 Nov 22;18(11):e0277150. doi: 10.1371/journal.pone.0277150 (PMC10664926; doi:10.1371/journal.pone.0277150)
Supplement: S3 Table — Incidental results by study site and season for field studies during detection trials conducted at the study sites from June 27, 2021, through July 14, 2022. (DOCX) [file pone.0277150.s004.docx]

**S4 Table. Incidental detection trial results.** Incidental results by study site and season for field studies during detection trials conducted at the study sites from June 27, 2021, through July 14, 2022.

| **Study Site** | **Season** | **Decoys Placed** | **Decoys Available** | **Decoys Found** | **Detection Probability** |
| --- | --- | --- | --- | --- | --- |
| **Frontier I** | Spring | 40 | 34 | 8 | 0.24 |
| **Marble River** | Spring | 41 | 31 | 24 | 0.77 |
| **Mountain Wind I and II** | Spring | 64 | 64 | 36 | 0.56 |
| **Pinyon Pines I and II** | Spring | 48 | 47 | 14 | 0.30 |
| **Shiloh I** | Spring | 47 | 47 | 17 | 0.36 |
| **Wild Horse** | Spring | 48 | 48 | 19 | 0.40 |
| **Frontier I** | Summer | 27 | 15 | 6 | 0.40 |
| **Marble River** | Summer | 32 | 23 | 13 | 0.57 |
| **Mountain Wind I and II** | Summer | 32 | 32 | 24 | 0.75 |
| **Pinyon Pines I and II** | Summer | 36 | 36 | 16 | 0.44 |
| **Shiloh I** | Summer | 64 | 64 | 55 | 0.86 |
| **Wild Horse** | Summer | 64 | 63 | 15 | 0.24 |
| **Frontier I** | Fall | 64 | 52 | 29 | 0.56 |
| **Marble River** | Fall | 43 | 32 | 30 | 0.94 |
| **Mountain Wind I and II** | Fall | 48 | 48 | 31 | 0.65 |
| **Pinyon Pines I and II** | Fall | 60 | 58 | 19 | 0.33 |
| **Shiloh I** | Fall | 32 | 31 | 22 | 0.71 |
| **Wild Horse** | Fall | 32 | 32 | 5 | 0.16 |
| **Frontier I** | Winter | 46 | 39 | 20 | 0.51 |
| **Marble River^a^** | Winter | 0 | 0 | 0 | 0 |
| **Mountain Wind I and II^a^** | Winter | 0 | 0 | 0 | 0 |
| **Pinyon Pines I and II** | Winter | 48 | 47 | 10 | 0.21 |
| **Shiloh I** | Winter | 48 | 44 | 21 | 0.48 |
| **Wild Horse** | Winter | 32 | 31 | 10 | 0.32 |

^a^Winter trials were not conducted at Marble River or Mountain Wind I and II due to the amount of snowfall and limited site access
