# Supplementary material for: Incidental eagle carcass detection can contribute to fatality estimation at operating wind energy facilities
Source: PLoS One. 2023 Nov 22;18(11):e0277150. doi: 10.1371/journal.pone.0277150 (PMC10664926; doi:10.1371/journal.pone.0277150)
Supplement: S4 Table — Trial results by activity being performed by Operations and Maintenance staff at the time of detection during the detection trials conducted at the study sites from June 27, 2021, through July 14, 2022. (DOCX) [file pone.0277150.s005.docx]

**S5 Table. Incidental detection trial results by Operations and Maintenance staff activity.**

Trial results by activity being performed by Operations and Maintenance staff at the time of detection during the detection trials conducted at the study sites from June 27, 2021, through July 14, 2022.

| **Study Site** | **Maintenance** | **Driving** | **Land Management** | **Turbine Inspections** | **Surveying** | **Total Reported** | **Total Found** |
| --- | --- | --- | --- | --- | --- | --- | --- |
| **Frontier I** | 12 | 0 | 0 | 0 | 0 | 12 | 63 |
| **Marble River** | 12 | 3 | 10 | 2 | 0 | 27 | 67 |
| **Mountain Wind I and II** | 14 | 41 | 1 | 32 | 0 | 88 | 91 |
| **Pinyon Pines I and II** | 27 | 13 | 0 | 1 | 0 | 41 | 59 |
| **Shiloh I** | 11 | 26 | 0 | 68 | 0 | 105 | 115 |
| **Wild Horse** | 15 | 13 | 0 | 3 | 3 | 34 | 49 |
| **Total** | **91** | **96** | **11** | **106** | **3** | **307** | **444** |
