# Supplementary material for: Incidental eagle carcass detection can contribute to fatality estimation at operating wind energy facilities
Source: PLoS One. 2023 Nov 22;18(11):e0277150. doi: 10.1371/journal.pone.0277150 (PMC10664926; doi:10.1371/journal.pone.0277150)
Supplement: S5 Table — Results using AICc for 18 models of incidental detection using 918 decoy placements during detection trials conducted at 6 study sites from June 27, 2021, through July 14, 2022. The term (1|Site) represents a random effect for site; all other effects are fixed. Originally, there were 64 different models; however, when multiple models resulted in the same AICc value (and thus were different parameterizations of the same underlying model), we only included one model in the table below for comparison and interpretation. (DOCX) [file pone.0277150.s006.docx]

**S6 Table. Model selection results for incidental detection.** AICc model scores for 18 distinct models of incidental detection using 918 decoy placements during detection trials conducted at 6 study sites from June 27, 2021, through July 14, 2022. The term (1|Site) represents a random effect for site; all other effects are fixed. Originally, there were 64 different models; however, when multiple models resulted in the same AICc value (and thus were different parameterizations of the same underlying model), we only included one model in the table below for comparison and interpretation.

| **Fixed and Random Effects** | **Number of Parameters** | **AICc^a^** | **Δ AICc^b^** |
| --- | --- | --- | --- |
| Density Quartile+Season:Viewshed Complexity + (1\|Site) | 16 | 998.74 | 0.00 |
| Viewshed Complexity+Density Quartile + (1\|Site) | 7 | 1001.79 | 3.05 |
| Season:Viewshed Complexity+Viewshed Complexity:Density Quartile + (1\|Site) | 22 | 1002.81 | 4.07 |
| Density Quartile+Viewshed Complexity:Density Quartile + (1\|Site) | 13 | 1004.02 | 5.28 |
| Season+Viewshed Complexity+Density Quartile + (1\|Site) | 10 | 1004.05 | 5.31 |
| Season+Viewshed Complexity:Density Quartile + (1\|Site) | 16 | 1007.07 | 8.33 |
| Season:Viewshed Complexity+Season:Density Quartile + (1\|Site) | 25 | 1012.87 | 14.13 |
| Season:Viewshed Complexity+Season:Density Quartile+Viewshed Complexity:Density Quartile + (1\|Site) | 31 | 1016.37 | 17.63 |
| Viewshed Complexity+Density Quartile+Season:Density Quartile + (1\|Site) | 19 | 1017.38 | 18.64 |
| Viewshed Complexity+Season:Density Quartile+Viewshed Complexity:Density Quartile + (1\|Site) | 25 | 1019.29 | 20.55 |
| Season:Viewshed Complexity + (1\|Site) | 13 | 1031.60 | 32.86 |
| Viewshed Complexity + (1\|Site) | 4 | 1034.08 | 35.34 |
| Season+Viewshed Complexity + (1\|Site) | 7 | 1036.13 | 37.39 |
| Season+Density Quartile + (1\|Site) | 8 | 1132.74 | 134.00 |
| Density Quartile + (1\|Site) | 5 | 1141.41 | 142.67 |
| Season+Season:Density Quartile + (1\|Site) | 17 | 1142.68 | 143.94 |
| Season + (1\|Site) | 5 | 1180.29 | 181.55 |
| (1\|Site) | 2 | 1188.92 | 190.18 |

^a^AICc is corrected Akaike’s Information Criterion.

^b^Δ AICc is the difference between the two models being compared.
